# Leprosy Neuropathy in a Non-Endemic Area: A Clinical and Pathological Study

**DOI:** 10.3390/biomedicines11092468

**Published:** 2023-09-06

**Authors:** Stefano Cotti Piccinelli, Matteo Tagliapietra, Tiziana Cavallaro, Beatrice Labella, Barbara Risi, Filomena Caria, Simona Damioli, Loris Poli, Alessandro Padovani, Sergio Ferrari, Massimiliano Filosto

**Affiliations:** 1 Department of Clinical and Experimental Sciences, University of Brescia, 25100 Brescia, Italy; stefano.cottipiccinelli@centrocliniconemo.it (S.C.P.); beatrice.labella93@gmail.com (B.L.); barbara.risi@centrocliniconemo.it (B.R.); alessandro.padovani@unibs.it (A.P.); 2 NeMO-Brescia Clinical Center for Neuromuscular Diseases, Guusago, 25064 Brescia, Italy; filomena.caria@centrocliniconemo.it (F.C.); simona.damioli@centrocliniconemo.it (S.D.); 3 Neurology Unit, Department of Neurosciences, Biomedicine and Movement Sciences, 37100 Verona, Italy; matteo.tagliapietra@aovr.veneto.it (M.T.); tiziana.cavallaro@aovr.veneto.it (T.C.); sergio.ferrari.298@gmail.com (S.F.); 4 Unit of Neurology, ASST Spedali Civili, 25100 Brescia, Italy; loris.poli@asst-spedalicivili.it

**Keywords:** sensory polyneuropathy, leprosy, pure leprosy neuropathy, multiple mononeuropathy

## Abstract

The extent of nerve involvement in leprosy is highly variable in distribution and clinical presentation. Mononeuropathies, multiple mononeuropathies, and polyneuropathies can present both in the context of a cutaneous and/or systemic picture and in the form of pure neuritic leprosy (PNL). The differential diagnosis of leprosy neuropathy remains challenging because it is a very rare condition and, especially in Western countries, is often overlooked. We report one case of the polyneuropathic form of PNL (P-PNL) and one case of multiple mononeuropathy in paucibacillary leprosy. In both cases, the diagnosis was achieved by performing a sural nerve biopsy, which showed subverted structure, severe infiltration of inflammatory cells in nerve fascicles, granulomatous abnormalities, and the presence of alcohol-acid-resistant, Ziehl–Neelsen-positive bacilli inside the nerve bundles. Leprosy remains an endemic disease in many areas of the world, and globalization has led to the spread of cases in previously disease-free countries. In this perspective, our report emphasizes that the diagnostic possibility of leprosy neuropathy should always be taken into account, even in Western countries, in the differential diagnostic process of an acquired sensory polyneuropathy or multineuropathy and confirms that nerve biopsy remains a useful procedure in working up neuropathies with unknown etiology.

## 1. Introduction

Leprosy is an infectious disease known for over three thousand years that affects mainly peripheral nerves and skin but also eyes, mucous membranes, bones, and testes. It is caused by *Mycobacterium leprae*, an intracellular, pleomorphic, acid-fast, aerobic bacterium with preferential trophism for Schwann cells and macrophages [1,2,3].

In 2018, more than 200.000 new cases were reported worldwide, with a prevalence of 0.25 per 10,000 population [4]. About 80% of the reported global new cases come from developing countries, mainly India, Brazil, and Indonesia [4,5]. Nowadays, in Europe, it is considered a rare disease, mainly linked to migratory phenomena. In Spain, between 2003 and 2013, 168 leprosy cases were registered: 24.6% were in Spanish patients, while 76.2% occurred in legally resident immigrants [5]. In Italy, data from the official leprosy register showed 12 Italian cases diagnosed between 1990 and 2009 but also a concurrent increase in imported incidence [6].

*Mycobacterium leprae* spreads with nasal secretions and skin contact from untreated multibacillary cases of leprosy and may locate on either the nasal or skin epithelia for initiating infection [7,8]. 

The incubation period is variable and may range from a few months to over 20 years [7].

The classical classification drawn up by Ridley and Joplin in 1966 is based on clinical, histological, and immunological criteria and divides leprosy into different groups: tuberculoid (T), borderline (B), and lepromatous (L), while the indeterminate form was added lately [9]. In 1982, the WHO added a classification of multibacillary and paucibacillary forms based on the number of bacilli present in the host [10]. In T-form, the patient shows single skin lesions or a small number of asymmetric lesions characterized by erythematous plaques. Damage to skin nerve fibers causes alopecia, anhidrosis, and a significant change in sensitivity: first thermal, then tactile, and pain sensitivity is lost. Thickening of the peripheral nerve sheath is common [11]. The B-form is the most common form and may present with a very broad clinical spectrum, including the Borderline Tuberculoid (BT) and the Borderline Lepromatous (BL) forms [9,12]. Usually, patients have multiple and severe peripheral nerve involvement [11,12,13,14].

The L-form is characterized by an ineffective cellular-mediated response to *Mycobacterium leprae*, which possibly leads to the hematogenous spread of the bacilli. Infiltration of the facial skin gives a typical clinical aspect known as “facies leonine” [9,12,13]. The peripheral nerves are enlarged, and impaired sensation may occur, leading to great disability [11]. Liver, spleen, adrenal glands, bone marrow, mucosal, and testicular involvement may be a consequence of the hematogenous spread of the bacteria [15]. A further type of leprosy is called pure neuritic leprosy (PNL), which accounts for about 3% of leprosy cases and is characterized by isolated single or multiple peripheral nerve involvement [16]. The most common presentations are mononeuropathy (79%) and multiple mononeuropathy (10.5%) [17,18,19]. A polyneuropathic form (P-PNL) is rarely reported in the literature, and diagnosis is often missed, especially in Western countries, where leprosy is not an endemic disease [20,21,22,23,24].

Exposure to the bacillus is not sufficient for the disease to develop; some factors, such as nutrition, hygiene, and immune response, seem to play an important role [9]. The form developed by each individual mainly depends on the host’s immune capacity to fight *Mycobacterium leprae*; in general, patients with the T-form have a good ability to respond to Mycobacterium, while anergic subjects develop the L-form [14,25].

Multiple genes may influence the risk of contracting leprosy and modulate the clinical manifestation of the disease, according to several twin studies, segregation analysis, family-based linkage studies, association studies, and genome-wide investigations [26]. The lymphotoxin gene, the *PARK2/PCRG* gene regulatory region, the *TLR1* gene, and the *NOD2* gene are all among the most studied genes and have compelling evidence supporting their modulatory roles [27,28,29,30].

Here we report two cases of leprosy neuropathy with different clinical expressions diagnosed in Italy and describe their clinical and pathological findings, emphasizing the challenging diagnostic process.

## 2. Case Presentation

### 2.1. Case 1

A 33-year-old Brazilian woman who has lived in Italy for 6 years with no history of traveling back to her country came to our clinic complaining about sensory impairment involving both hands and feet for about 3 years. She described her symptoms as pins and needles, pricking, burning, tingling, and numbing sensations associated with a reduction in tactile and pain sensitivity.

Symptoms, early appearing in a fluctuating way, were constantly present over the last 8 months and slightly worsening over time. She weighs 47 kg with a body mass index of 20.8. She is unemployed and has smoked five cigarettes per day for 10 years. She has two healthy children and no history of alcohol or drug abuse.

No family history of neurological disease was reported. In her medical history, she has been suffering from mild hypothyroidism for 10 years and was treated with 75 micrograms of levothyroxine per day.

Neurological examination showed symmetrical tactile and stocking pin-prick impairment in both legs below the knee and hands with glove distribution, vibratory and proprioception sensory loss in the lower limbs and hands, and absent ankle jerk tendon reflexes. No other neurological signs were present. The general physical examination was normal; specifically, no skin changes or cardiac, pulmonary, or abdominal abnormalities were noted.

Routine laboratory tests (including blood cell count, blood glucose, vitamin B12, and folate), immunological tests (including anti-nuclear and anti-extractable nuclear agents antibodies, anti-neutrophil cytoplasmic antibodies, Lupus anticoagulant, C-reactive-protein), angiotensin-converting enzyme, Hepatitis B surface antigen, anti-Hepatitis C antibodies, anti-HIV antibodies, anti-Borrelia Burgdorferi antibodies, anti-Treponema antibodies, anti-transglutaminase antibodies, tumor markers, antibody IgG and IgM to gangliosides GM1, GM2, GD1a, GD1b, and IgM to myelin-associated glycoprotein (MAG) yielded normal findings.

Cerebrospinal fluid (CSF) examination showed a normal amount of protein and cells, no neoplastic cells or oligoclonal bands, and a normal link index.

The transthyretin (TTR) gene was analyzed, and no mutations were found.

The electroneurographic study (ENG) showed the absence of sensory action potential (SAP) in the sural, median, and ulnar nerves. The amplitude of the motor action potential (MAP) of the right peroneal nerve was reduced (1.60 mV), while the MAPs of the left peroneal nerve and median and ulnar nerves were within normal range. Needle electromyography (EMG) showed no spontaneous activity with scattered motor unit potential (MUP) with increased amplitude.

**Figure 1:**
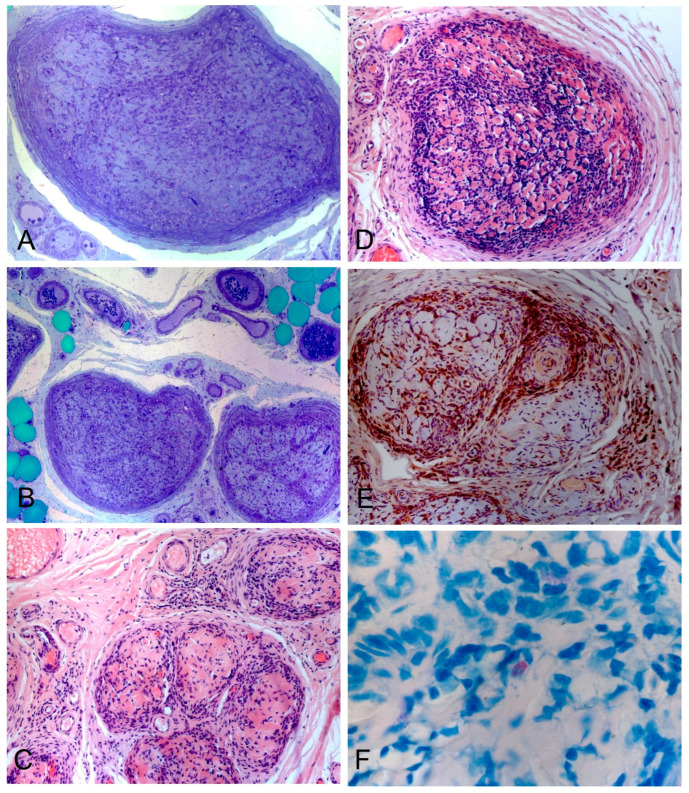
Pathological aspects of sural nerve biopsy in patient 1. (**A**,**B**) Semithin sections stained with basic toluidine blue: A subversion of the structure of the nervous fascicle is observed: myelinated fibers are not recognizable in the endoneurium, which has a high cellularity. There is active endo- and perineural perivascular infiltration, in particular (**B**) of the subperineural space and the endoneurial septa. (**C**,**D**,**E**) Hematoxylin-eosin staining: infiltration of the perineurium is observed. The accumulations of cells have lymphocytic and macrophagic morphology; some of these have a multivacuolated cytoplasm (foam cells), clearly evident with immunocytochemical characterization showing a clear preponderance of macrophage cells, CD68+ (**C**). (**F**) Ziehl–Neelsen staining shows rare cells with intracytoplasmic acid-resistant material.

In order to achieve a diagnosis, a sural nerve biopsy was performed. Histological analysis showed nerve fascicles with a subverted structure, the absence of nerve fibers in the endoneurium, and severe infiltration of inflammatory cells (Figure 1A–E). Alcohol-acid-resistant, Ziehl–Neelsen positive bacilli inside the nerve bundles were identified, thus supporting the diagnosis of leprosy neuropathy (Figure 1F).

The patient was referred to the National Leprosy Referral Center and treated with Dapsone and Rifampicin.

At 6-month follow-up, the patient was clinically stable with no progression of the sensory deficit that was still present. The electroneurographic study was unchanged.

### 2.2. Case 2

A 44-year-old Italian male presented with long-lasting ankle and elbow arthralgia, later associated with a diffuse rash of non-tender, non-itchy polycyclic annular plaques with acral prevalence. Medical and familial history was unremarkable. Routine laboratory and immunology tests were normal. In the suspicion of psoriatic arthritis, 25 mg daily oral prednisone was introduced with initial clinical remission, followed by recurrence upon tapering. Add-on therapy with methotrexate at five months and secukinumab at nine months failed to relieve steroid dependence.

Antibiotic treatment with amoxicillin-clavulanate for an intercurrent productive cough resulted in rapid deterioration with hyperpyrexia and swelling of the extremities, followed by sensory impairment in the left foot. A biopsy of the skin lesion was obtained and showed sarcoid-like abnormalities with superficial and deep dermal granulomatous infiltration, not supportive of psoriasis. Secukinumab was discontinued.

In the following year, repeated immunological testing was unremarkable. Normal chest computed tomography, tracheobronchoscopy, breakup time, Schirmer testing, and angiotensin-converting enzyme dosage ruled out sarcoidosis. Duodenal biopsy and polymerase chain reaction (PCR) for *T. whipplei* in body fluids were inconclusive. A second biopsy of a skin lesion demonstrated non-caseating epithelioid granulomas and concurrent subepidermal lichenoid-like dermal plexiform lymphoplasmacytoid infiltrates. Staining for microorganisms was negative (periodic acid Schiff–Alcian Blue, Grocott–Gömöri methenamine silver, Gram, Warthin–Starry, and Ziehl–Neelsen); however, several granular bodies of uncertain significance were observed within the granulomatous lesions on anti-Treponema immunohistochemical staining.

Steroids were discontinued but soon reintroduced upon relapse. Etanercept 50 mg weekly was added to the methotrexate but was soon abandoned by the patient for perceived ineffectiveness in favor of a steroid-only therapy. At 31 months, the patient reported hand paresthesia, abolished tactile sensation of the soles, and a painless ulceration of the left sole, prompting a neurological referral.

Neurological examination showed severe impairment in superficial tactile, pain, and thermal sensation in the lateral side of the left foot (sural nerve area), in the medial side of the left lower leg and foot (saphenous nerve area), and in the right foot as a whole. On the other hand, a mild decrease in vibration perception at the great toe and ankle was observed. Gait, proprioception, deep tendon reflexes, and limb strength were normal.

ENG showed the absence of SAPs of the left superficial peroneal, left dorsal branch of the ulnar, and both sural nerves, as well as a decrease in the right dorsal branch of the ulnar nerve and left median nerve SAP amplitudes. Peroneal and tibial MAPs were bilaterally absent, and ulnar nerve MAPs were at the lower limit of normal amplitude; instead, the median nerve was preserved bilaterally. Spontaneous activity was observed in the distal lower limb muscles, associated with a decrease in recruitment. The sympathetic skin reflex was barely elicitable at the palms and absent at the soles.

Upon further questioning, the patient disclosed a two-month-long stay in high-incidence areas in Brazil 15 years earlier.

**Figure 2:**
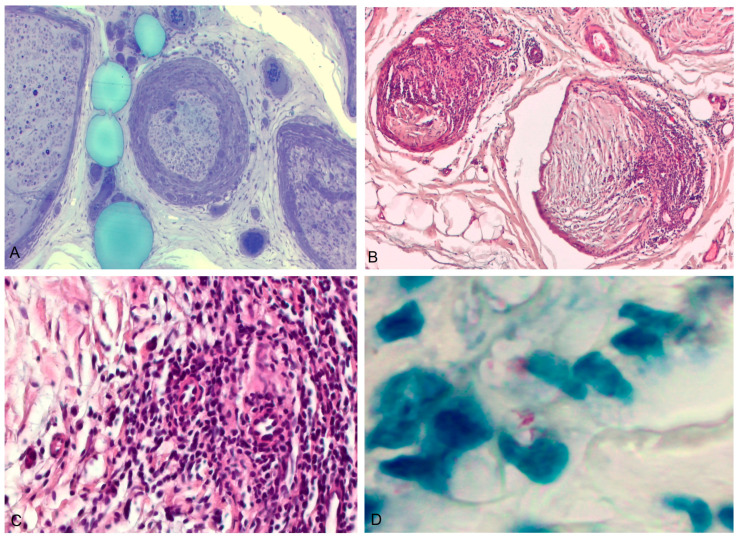
Pathological aspects of sural nerve biopsy in patient 2. (**A**) Toluidine blue stain and (**B**) hematoxylin-eosin: Sural nerve biopsy shows alterations of the nerve fascicles with thickening and infiltration of perineurium and formation of granulomas. (**C**) Hematoxylin-eosin: the presence of granulomas. (**D**) Ziehl–Neelsen stain shows rare acid-alcohol-resistant particles in nerve fascicles.

A nerve biopsy of the left sural nerve revealed a dramatic, asymmetric peri- and endoneurial granulomatous infiltration associated with endoneurial foam cells (Figure 2A–C). Interspersed between the lesions, scarce bacillary alcohol-acid-resistant bodies were observed on Ziehl–Neelsen staining (Figure 2D). *M. Leprae* DNA was detected by PCR in the same specimen. The diagnosis was later confirmed by skin smear testing at the National Leprosy Referral Center, with the discontinuation of immunosuppressive therapy and the introduction of specific antimicrobial treatment with benefit. 

## 3. Discussion and Conclusions

Peripheral polyneuropathy, even though it is a frequent neurological complaint, remains a diagnostic challenge. Heterogeneity in etiology, distribution, pathology, and severity makes it difficult to achieve a correct diagnosis, and many patients remain undiagnosed despite exhaustive workup [31]. In a case series from Italy, one-fifth (19%) of patients with chronic neuropathies remained idiopathic [32].

The patients described here present, in the first case, a chronic distal and symmetric axonal sensory polyneuropathy without skin lesions or systemic involvement that has undergone extensive investigation before reaching the correct diagnosis and, in the second case, a predominantly sensory multineuropathy with an arthritic-like presentation resembling a rheumatological disorder and associated with skin lesions that have not been well identifiable for a long time.

The first case can be classified as a P-PNL as there is a polyneuropathic distribution with no cutaneous or systemic signs.

PNL was defined as a type of leprosy without skin lesions, enlargement of large nerve trunks, and sensory loss in the area of its distribution [24]. As a distinctive type of leprosy, it has always been an enigma, as clinical and management ambiguities remain. Nonetheless, there is worldwide acceptance of PNL as a distinct type of leprosy [23,24]. An epidemiological study carried out in India identified 65 (4.2%) PNL out of the total 1542 leprosy patients seen over the 1993 to 2003 period [33]. In Brazil, among 2225 patients diagnosed with leprosy between 1998 and 2016, 175 cases of PNL were diagnosed [24]. Most of the PNL cases are mononeuropathies or multiple mononeuropathies and were observed in eastern and tropical countries [17,21,23,24]. Hyperesthesia and anhidrosis may occur when small cutaneous nerve fibers are involved [17,34,35]. Cranial nerves are involved in 18% of cases [18].

Only a few reports on P-PNL are available in the literature [17,19,21,36]. It is usually distal and symmetrical with proprioception, light touch, temperature, and pain sensory abnormalities without muscle weakness [17,19,21]. Tendon reflexes can be lost, reduced, or preserved, and electroneurography may show both demyelinating and axonal involvement or may be normal, thus suggesting the involvement of small fibers [17,19,20,21].

The second case can be classified as multiple mononeuropathy in paucibacillary leprosy.

Polyarthritis, skin lesions, and transient steroid responsiveness are frequently reported in leprosy, especially during acute lepra reactions, but are easily misinterpreted in non-endemic countries, where autoimmune rheumatologic conditions are far more common [37]. Multiple mononeuropathy is not enough to establish a diagnosis because a similar neuropathic involvement is relatively common in vasculitis, rheumatoid arthritis, scleroderma, and granulomatous disorders such as sarcoidosis [37,38]. Multiple mononeuropathies have also been linked to a number of immunosuppressive drugs, including TNF inhibitors (etanercept, infliximab, and adalimumab) [39,40,41]. The prevalent involvement of small nerve fiber modalities (autonomic dysfunction, pain, and abnormal thermal sensation) compared to large fiber involvement (abnormal proprioception, vibratory sensation, deep tendon reflexes, and motor function) could help in suspecting leprosy neuropathy. However, it is not pathognomonic in any way [36,42].

Finally, the diagnosis of leprosy neuropathy remains challenging because it is a very rare condition and, especially in Western countries, is often overlooked.

In both our cases, the ultimate diagnosis was possible only by doing a sural nerve biopsy, which, therefore, remains a valuable diagnostic tool for investigating apparently idiopathic peripheral axonal neuropathies. However, its sensibility is reported at around 75%, and it needs to be evaluated by expert personnel [36]. Nerve histological abnormalities may not be specific and range from perineural thickening, perineural and endoneurial inflammation to granuloma formation both in endoneurial and perineurial locations, foam cell infiltration, occasional necrosis and vasculitis, and myelin and axonal loss [31]. Since evidence of the bacilli may be missed, it must be clear that their absence in the biopsy samples does not rule out leprosy neuropathy. As a result, when there is a predominantly sensory chronic progressive neuropathy with an unknown etiology, this condition should be taken into consideration, and additional testing, such as a skin biopsy, should be carried out.

Despite the global decline in infections, leprosy is still an endemic disease in many areas of the world, and globalization has led to the spread of cases in countries previously considered disease-free. Our cases should be a reminder to clinicians to keep in mind the diagnostic possibility of Leprosy in the diagnostic workup of peripheral neuropathies.

## Data Availability

Data are available upon reasoned request.

## Availability of Data and Material

Data sharing is not applicable to this article as no datasets were generated or analyzed during the current study.

## Abbreviations

T	Tuberculoid
B	Borderline
BT	Borderline Tuberculoid
BL	Borderline Lepromatous
CSF	Cerebrospinal fluid
EMG	Electromyography
ENG	Electroneurographic study
L	Lepromatous
MAP	Motor action potential
MUP	Motor unit potential
MAG	Myelin-associated glycoprotein
PCR	Polymerase chain reaction
P-PNL	Polyneuropathic form of PNL
PNL	Pure Neuritic Leprosy
SAP	Sensory action potential
TTR	Transthyretin
